# Editorial: Proteoglycans in the Tumor Microenvironment

**DOI:** 10.3389/fonc.2022.872417

**Published:** 2022-04-07

**Authors:** Jlenia Brunetti, Manuela Viola, Federico Corti

**Affiliations:** ^1^ University of Siena, Department of Medical Biotechnology, Siena, Italy; ^2^ University of Insubria, Department of Medicine and Surgery, Varese, Italy; ^3^ Yale University School of Medicine, Department of Internal Medicine, New Haven, CT, United States

**Keywords:** proteoglycans, extracellular matrix (ECM), immunotherapy, matrisome, disease marker

The Proteoglycans (PGs) family consists of core proteins covalently attached to glycosaminoglycans (GAGs) belonging to different classes: heparan sulfates/heparin, chondroitin/dermatan sulfates, and keratan sulfate. A last family of GAGs includes the molecule hyaluronan, which is the only one not bound to a protein core nor chemically modified by sulfates. Even if hyaluronan is a unique chemical structure, it can be present within the extracellular matrix (ECM) at various molecular weights with different biological activity.

PGs are ubiquitous, and, in each tissue or cell compartment, they play crucial roles in promoting cell signaling and migration by interaction with growth factor and/or their receptors, intracellular enzymes, extracellular ligands, matrix components, inflammatory cytokines, and ECM structural proteins. Besides regulating the normal behavior and turnover of tissues, PGs can also mediate tumor-microenvironment interactions.

The composition of the ECM and, even more importantly, its modification through altered genetic/epigenetic expression and activation/inhibition of degrading enzymes can be the key factors that favor or restrain the growth and migration of cancers. Because of the involvement in each aspect of cancers metabolism, the same PGs can be either investigated as markers for the disease prognosis or as targets for new therapies.

PGs are indeed the perfect protagonists of precision medicine; in fact, “the single marker for a type of cancer is seldom resolutive or decisive and groups of markers are more informative”; in this light, the HSPGs (which share carbohydrate chain synthetic enzymes, probably the regulation system and that often undergo shedding processes) are interesting candidates, as affirmed Furini and Falciani. In their review “Expression and Role of Heparan Sulfated Proteoglycans in Pancreatic Cancer” Furini and Falciani confronted and discussed the expression of several synthetic enzymes using data from other authors’ papers by means of a comparative data and search engine tool. The use of such resources is becoming more and more important in the prediction of which marker should be evaluated for each cancer type and will help MDs tailor treatment towards individual patients and help researchers to better direct the development of new antitumoral drugs and strategies.

One of the promising strategies against cancer is immunotherapy, which is highly dependent on the patient immune system as well as on the inflammation status of the target tissue. Effectively, the matrisome, a growing set of matrix and matrix-related molecules, is an intriguing and very promising target for blocking cancers, even though the main difficulty is due to the ubiquitous presence of the molecules and the high individual variability. The tumor microenvironment has been reported as a major predictor of immunotherapy success; for this reason, being able to modulate the immune response of each patient can be very important for the outcome of any anticancer therapy. The knowledge of the biology of the matrisome is therefore pivotal, as underlined by Hirani et al. In particular, Hirani et al. explore the role of versican because of the multiple variants present in tissues and of its binding with several other matrix molecules. The authors conclude with a specific reference to the polysaccharide moiety of versican; this is a chondroitin sulfate chain that may provide the most specific targeting. Nevertheless, the GAGs are particularly difficult structures to study and correlate with cancer progression and the extracellular matrix can be an obstacle to immunotherapy since apart from modulating the behavior of the immune cells, it is itself remodeled by those cells. Therefore, the current therapies targeting the ECM molecule are at the same time intriguing yet unsatisfactory.

He et al. explored a number of major therapeutic candidates for the regulation of ECM related to cancer immunity and concluded in their review that “PEGPH20 (a recombinant human hyaluronidase), targeting Hyaluronan, and Cetuximab (a commercial monoclonal antibody) targeting collagen may have the potentiality to be explored, although targeting ECM has not yet yielded satisfactory results as an adjuvant immunotherapy”.

Degrading enzymes, such as hyauronidases or heparanase, play an important role in the re-organization of the ECM in order to make it permissive to the cancerous cell proliferation and migration beyond the escape from the immune system. Cell migration leads to one of the worst scenarios for a cancer patient: relapse due to metastasis. Also in this particular aspect, the ECM has a central role, both in facilitating the physical movement and in allowing the colonization of the new tissue.

An important PG in patients suffering from poorly prognostic malignancies with high relapse rates is Syndecan-1, whose metabolism is even more complicated by the possibility of different effects due to its shedding and nuclear translocation; in fact, those events further enhance Syndecan-1 modulation of the epithelial–mesenchymal transition as well as tumor invasiveness, as reported by the review of Guo et al. The possibility of different forms that are not identifiable using a gene expression approach directly on the target molecule explains the conclusion of all the papers of the topic: the ECM molecules are perfect targets both for their prognostic value in predicting the cancer outcome and for the modulation of the immune response. Nevertheless, the study of the changes in the ECM microenvironment should include the synthetic enzymes of the GAG moiety (the so-called “gagosome”) and the degrading enzymes (e.g., hyaluronidases, heparanases, etc.). Moreover, because of the high numbers of variables, the software resources already available must be implemented with more information and increased data numerousness.

## Author Contributions

JB managed the network duties; MV wrote the first draft of the manuscript; FC review and edited. All authors contributed to manuscript revision, read, and approved the submitted version

## Conflict of Interest

The authors declare that the research was conducted in the absence of any commercial or financial relationships that could be construed as a potential conflict of interest.

## Publisher’s Note

All claims expressed in this article are solely those of the authors and do not necessarily represent those of their affiliated organizations, or those of the publisher, the editors and the reviewers. Any product that may be evaluated in this article, or claim that may be made by its manufacturer, is not guaranteed or endorsed by the publisher.

